# Overview of the Analytes Applied in Genotypic HIV Drug Resistance Testing

**DOI:** 10.3390/pathogens11070739

**Published:** 2022-06-29

**Authors:** Hezhao Ji, Paul Sandstrom

**Affiliations:** 1 National Microbiology Laboratory at JC Wilt Infectious Diseases Research Centre, Public Health Agency of Canada, Winnipeg, MB R3E 3R2, Canada; paul.sandstrom@phac-aspc.gc.ca; 2 Max Rady College of Medicine, University of Manitoba, Winnipeg, MB R3E 0J9, Canada

**Keywords:** HIV, drug resistance, testing, analytes, specimens, performance

## Abstract

The close monitoring of HIV drug resistance using genotypic HIV drug resistance testing (HIVDRT) has become essential for effective HIV/AIDS management at both individual and population levels. Over the years, a broad spectrum of analytes or specimens have been applied or attempted in HIVDRT; however, the suitability and performance of these analytes in HIVDRT and the clinical relevance of the results from them may vary significantly. This article provides a focused overview of the performance, strengths, and weaknesses of various analytes while used in HIVDRT, which may inform the optimal analytes selection in different application contexts.

## 1. Introduction

Human immunodeficiency virus (HIV) and the acquired immunodeficiency syndrome (AIDS) it causes, pose significant public health concern at a global level. With the enhanced access and improved efficacy of antiretroviral therapy (ART), HIV/AIDS has become a manageable chronic reaction in resource-permitted settings [1]. ART improves the lives of people living with HIV, and it also plays a vital role in reducing HIV transmission and HIV incidence [2]. However, HIV drug resistance (HIVDR) significantly decreases the ART efficacy and undermines its benefits. Over 27.5 million people were receiving ART globally by the end of 2020, but up to 24% of pre-treatment patients and 50~90% of patients failing ART may harbor ART-resistant variants [3,4]. Among many recommendations from the 2017 World Health Organization (WHO) Global Action Plan on HIVDR are the expanded coverage and improved effectiveness of genotypic HIVDR testing (HIVDRT) for surveillance and clinical purposes [4]. Any strategy that promotes the access, affordability, sensitivity, and accuracy of HIVDRT would benefit HIVDR management globally. The appropriate selection of clinical specimen or analyte plays a vital role in the assay performance and subsequent HIVDR data interpretation and application. 

Current HIVDRTs examine the presence of drug resistance-associated mutation (DRM) either via allele-specific assays targeting specific viral mutation(s) or by HIVDR genotyping, which sequences the ART-targeted HIV-1 genes and analyzes all potential DRMs collectively. Any HIV-positive specimen or analyte that contains HIV ribonucleic acid (RNA) or deoxyribonucleic acid (DNA) for the target HIV gene(s) could be used for HIVDRT. Unsurprisingly, a broad spectrum of analytes has been applied or attempted in HIVDRT. Most of such analytes are also applicable for other HIV molecular assays, such as viral load (VL) determination. This HIVDR thematic article provides a focused overview of various analytes’ performance, strengths, and weaknesses while applied in HIVDRT. 

## 2. Varied Analytes for HIVDRT 

The commonly-used analytes are summarized as below: (Table 1).

### 2.1. Plasma

Plasma is the supernatant after the cellular components of the anti-coagulated blood are removed after centrifugation. Plasma specimens are usually collected for molecular assays such as HIVDR testing, which examines the HIV viral RNA it contains. Plasma has the highest viral RNA concentration among all analytes applied in HIVDRT. The viral population in plasma best represents the cell-free, replication-competent, circulating HIV viruses in the patients. HIV viral RNA in plasma remains stable in ambient temperature and long-term storage under freezing conditions [5,6,7]. 

Plasma is the gold standard analyte most commonly used for HIVDRT [8]. It serves all clinical, surveillance, and research HIVDRT needs. All breakthroughs in HIVDRT assay development and validation were first established using plasma and then applied to other analytes. The prevention of blood clotting maximizes the number of viral templates available in plasma for HIV genotyping. This is particularly important for specimens collected from ART-treated individuals whose VLs could be extremely low. It is noteworthy that ethylenediaminetetraacetic acid (EDTA), but not heparin, should be used as the anti-coagulant if a molecular assay such as HIVDRT is to be performed on the derived plasma. This is due to the inhibitory effect of heparin in the downstream polymerase chain reaction (PCR) amplifications [9,10].

### 2.2. Serum

Serum is the fluid left after whole blood is naturally clotted. The compositions of plasma and serum are nearly identical except for fibrinogen, which is present in plasma but naturally removed from serum during clotting. Serum is the most commonly used substitute for plasma in molecular HIV assays. While plasma is always the preferred analyte, serum is often used for serological HIV diagnosis. The suitability of serum versus plasma for HIVDRT testing varies largely depending on the availability of centrifugation devices and the usage of anti-coagulant during the sample collection. However, remnant frozen sera from diagnostic testing are often utilized in retrospective HIVDR surveillance and research projects [8]. 

While applied in HIV molecular assays, another notable difference between plasma and serum analytes is the concentration of HIV contents. A small portion of HIV particles or viral RNA can be trapped in the blood coagulum during clotting, rendering lower VLs in serum than in plasma [11,12]. There is no evidence showing different HIVDR profiles as determined using plasma or serum, implying that the HIV viral RNA loss resulting from clotting is non-selective. However, precautions should still be taken if serum specimens are used for HIVDRT and viral diversity analysis, especially when next generation sequencing (NGS) technologies are used. NGS resolves the intra-host viral diversity and DRMs of lower abundance with a significantly higher resolution than conventional population-based Sanger sequencing [13,14]. Moreover, serum might not be an ideal analyte for patients with lower expected VLs, such as those currently on ART.

The advantages of plasma and serum analytes in HIVDRT are apparent. However, their limitations are also evident. Both analytes require well-trained phlebotomists, skilled lab personnel, stringent cold-chain transportation, and low-temperature storage conditions to maintain the specimen quality and HIV template integrity. These requirements limit the suitability and feasibility of plasma and serum for applications in remote or resource-limited settings (RLS) [15,16]. Searching for ideal alternative analytes or specimen collection metrics has been an everlasting interest for HIVDR professionals.

### 2.3. Whole Blood

Besides plasma and serum, anticoagulated whole blood (WB) is another commonly-used laboratory analyte. WB is widely used when the isolation of plasma/serum is not feasible, but nucleic acid extraction from WB on time is doable. HIV RNA may retain its integrity for 72 h in WB at an ambient temperature of 25 °C [17]. Therefore, WB could be a suitable substitute for HIV molecular assays targeting HIV viral RNA. Despite this, most WB-based HIVDR studies employed only the DNA extracts.

While plasma and serum both contain HIV viral RNA primarily, the HIV-infected cellular components in WB carry HIV proviral DNA. Depending on the nucleic acid extraction strategies applied, the HIV genetic materials recovered from WB could include viral RNA, proviral HIV DNA, or a combination of both if total nucleic acid (TNA) is extracted. Likewise, the application values of the WB specimens vary depending on the HIV templates used in further HIVDRT. Using RNA extract from WB may approximate the results from plasma/serum reflecting the circulating HIV population. In contrast, data from the DNA extracts may convey the information from HIV proviruses, a distinct archival viral population that is not as informative for patient management.

Steegen et al. assessed the feasibility of HIVDRT using DNA extracted from WB and compared it with results from plasma viral RNA [18]. High genotyping success rates were achieved for all specimens with detectable viral loads from plasma viral RNA and DNA from WB. Moreover, HIV protease (PR) and reverse transcriptase (RT) genes were successfully amplified from 67.7% and 61.3% of WB DNA preparations from patients with undetectable plasma VL [18]. While the viral DNA from WB boosts the HIV amplification rates, HIVDR data from such DNA extracts were often discordant with RNA extracts, confirming that they reflect different viral populations [19]. In addition, HIVDR data from DNA extracts showed poor reproducibility, implying a possible founder effect [18]. Furthermore, defective proviruses that harbor stop codons in the HIVDRT target genes are not rare, and excluding such defective proviruses from the whole blood DNA-based HIVDR data would significantly improve its clinical application value [20].

To overcome the limitations of DNA from WB, using RNA or TNA from WB for HIVDRT may be beneficial. Saracino et al. demonstrated that combining viral RNA and DNA in HIVDR typing might help identify more DRMs in the patients and assist in a more informed, effective ART regimen selection [21]. Targeted extraction of the RNA or DNA components from WB by enzymatically removing the other is always an option if fewer confounding data are expected. However, this will inevitably reduce the net HIV application rates.

While WB sampling still requires phlebotomy, this analyte eliminates the need for centrifugation devices unavailable in many decentralized health facilities. Skipping the centrifugation step also reduces professional HIV exposure, artificial errors, or contaminations associated with plasma/serum sample processing. The relative stability of HIV RNA in WB at an ambient temperature also enables centralized lab testing if such specimens could be quickly transferred from the collection site to the testing lab, even in the absence of a cold chain [17].

### 2.4. Peripheral Blood Mononuclear Cells (PBMCs)

PBMCs consist of lymphocytes and monocytes isolated from the anti-coagulated whole blood by density gradient centrifugation. DNA extraction is usually performed on PBMCs to recover the cellular DNA containing proviral HIV DNA that is integrated into the HIV-infected cells’ genome. The derived DNA can then be used for HIVDRT. The genetic discordance between plasma HIV RNA and proviral HIV DNA from PBMCs has been well-documented [22,23,24,25]. Bi et al. showed that plasma viral RNA-based genotyping could detect HIV DRMs up to 425 days earlier than PBMC DNA when the plasma VL was less than 104 copies/mL [26]. It further highlights the slow turnover of the proviral population and the drastic distinction between the HIV proviruses and the circulating viral population in plasma [26]. A higher comparability of data from plasma and PBMCs was achievable only when the HIV duration is ≤2 years, the sample VLs are ≥5000 copies/mL, or when the patient is treatment naïve or off ART [27,28].

Depending on the ultimate HIVDRT objectives, proviral DNA from PBMC may have added value for comprehensive HIVDR profiling when HIV DRMs from HIV proviruses are considered [29]. PBMCs could be an alternative analyte for HIVDRT when using plasma viral RNA is not feasible or unsuccessful [30]. While conventional plasma RNA-based HIVDRT performs poorly on samples of low VLs, proviral DNA can be readily recovered from PBMCs in these patients for an extended period [31,32,33]. Therefore, PBMC may also satisfy the needs for retrospective HIVDR analysis or population-level surveillance, in which the order of DRM occurrence is less of a consideration.

Interestingly, Armenia et al. showed that, combined with low nadir CD4 counts and a short-term viral suppression history, PBMC-based HIVDR profiling could help predict the potential viral rebound after the ART regimen switch [34]. A bit counterintuitively, one recent study by Peng et al. reported that HIVDR mutants emerges in PBMS DNA months before they could be detected in plasma, suggesting that PBMC DNA could be an effective tool for early HIVDR detection [35]. Notably, this was from studying a single patient infected by HIV-1 CRF01_AE and experienced multiple ART failure episodes [35]. The validation of these findings in larger studies remains to be conducted. Moreover, Moraka et al. recently showed that HIV DRMs identified in PBMCs are often associated with defective proviral genomes among early-treatment children [36]. It could lead to an overestimated HIVDR profiling if such PBMC DNA-based HIVDR data were applied in patient care.

### 2.5. Dried Fluid Analytes

As a more affordable and practical sampling option, dried fluid analytes are increasingly applied in HIVDRT, especially in low- to middle-income countries where the HIV/AIDS pandemic hits the most but optimal sample collection and storage are not always feasible [37]. In such cases, dried fluid specimens collected/dried with different matrices or devices may be collected from peripheral clinics, community sites, or even self-collected from patients’ homes and then transferred under natural ambient conditions to laboratories for centralized testing.

HIV genetic materials in such dried analytes remain relatively stable over an extended period under a wide range of ambient temperatures and suboptimal shipping and storage conditions. However, the reduced assay sensitivity, consistency, and reproducibility due to the small sample volume and the inevitable RNA degradation are primary concerns when such dried analytes are applied in HIVDRT. Refining the preparation of such specimens, improving the integrity of the HIV templates they contain, and boosting the analytical sensitivity of such analytes for HIVDRT are all everlasting topics in this field of work.

Several dried fluid analytes that have been applied in HIVDR studies thus far are overviewed below. This list is by no means exhaustive, and more developments in this field should come up in the foreseeable future (Table 1).

#### 2.5.1. Dried Filter Paper Analytes (DFPAs)

DFPAs have been applied in diagnostic tests for decades, mainly due to the low cost and the ease of sample collection, transportation, and storage. The use of filter paper for blood collection dates back to the early 1960s, when dried blood spots (DBS) were first used for phenylketonuria diagnosis in pediatric patients [38]. Since then, filter paper has been used as a collection matrix for different body fluids, and DFPAs have been used for a broad spectrum of laboratory assays.

Depending on the fluids used, DFPAs for HIVDRT consist of conventional DBS, dried plasma spots (DPS), and dried serum spots (DSS). While the integrity of HIV templates in DFPAs inevitably decreases, HIVDRT with such analytes has often been reported, although their performance varies significantly [37,39].

#### DBS

DBS is the foremost DFPA option for HIVDRT. DBS is prepared by spotting and drying whole blood onto absorbent filter paper cards [40,41]. The blood could be from phlebotomy or a simple lancet-prick that even patients themselves can do. The small sample volume it requires (50~75 μL per spot) and no stringent need for phlebotomy make DBS an attractive option peculiarly for pediatric patients. The technical, practical, and operational advantages of DBS are evident. DBS can be naturally dried, packed, shipped in a regular envelope and stored at ambient temperature using zip-lock plastic bags with desiccant for days to weeks before processing. It helps avoid the requirement for cold-chain transportation while posing minimal biohazard peril to the ambience.

The performance of DBS in HIVDRT has been well-documented in varied contexts, and DBS is proven to be an accountable analyte for HIVDRT in most cases. Well-established DBS-based HIVDRT guidelines have been implemented [16]. With the proven success of DBS usage in HIVDRT from many studies, DBS is currently the WHO-recommended blood sampling method in low- to middle-income countries [16,42,43].

Although DBS is considered a suitable alternate analyte for HIVDRT, DBS has its intrinsic limitations. Like WB, the presence of proviral DNA in DBS renders a higher success rate for HIV amplification than DPS or DSS. However, such proviral DNA contribution also limits its capacity to manifest the HIVDR status of circulating replication-active HIV viruses. Studies have shown that proviral DNA in the DBS may contribute to up to 80% of the application success rates of these samples [44,45]. Steegen et al. successfully genotyped HIV PR and RT genes from 54.8% and 58.1% DBS DNA preparations from patients with undetectable plasma VL [18]. The comparability of DNased-treated extracts from DBS and DPS in HIV PCR success rates further confirms the proviral DNA contribution to the DBS-based HIV genotyping [45]. HIVDR profiling data from DBS and matching plasma are concordant only when the VL is ≥5000 copies/mL, and/or the patients have no ART experience, and/or the duration of HIV infection is short [27]. This restricts the DBS application in clinical HIVDR monitoring, for which low VL specimens from ART-treated patients are unavoidable.

DBS has been applied primarily in HIVDR surveillance and research studies. Occasionally, DBS was assessed for centralized HIVDR monitoring in which DBS specimens were collected from RLS where clinical monitoring is required, but DBS is the only feasible sampling option. Regardless of the success rate of HIVDRT in such studies, DBS is not an ideal option for HIVDR monitoring purposes. However, one exception is for HIV-infected pediatric patients, from whom collecting large blood volume via venipuncture is often impractical and unrealistic. With nearly half of the HIV-infected infants/children carrying HIVDR variants even before ART initiation in Sub-Saharan Africa, where HIV/AIDS hits the hardest, the advantage of DBS could be of particular significance in this patient category [3].

#### DPS

By spotting and drying plasma drops onto a filter paper card, DPS can be prepared similar to DBS. Rottinghaus et al. compared the performance of DBS and DPS against plasma in HIV VL determination and HIVDRT. Their data showed that DBS, not DPS specimens, rendered VL readouts comparable to plasma, and that DPS had a significantly lower HIVDRT success rate as compared to DBS (38.9% vs. 100%) for specimens with VL ≥1000 copies/mL [46]. The high concordance of VL determination and PCR amplification results between DPS and DNase-treated DBS specimens confirms the proviral DNA contribution to the DBS-based HIVDR data [45]. It also necessitates a shorter storage time, a lower ambient temperature, and a shorter transportation for DPS than DBS due to reduced RNA stability [47].

While DPS consistently produces lower VL values, DPS-derived HIVDR data are highly comparable with those derived from plasma of the same patients [39], implying that the RNA degradation-induced HIV template loss in DPS is non-selective and HIVDR variants are not affected disproportionally. It makes DPS a better option than DBS for clinical HIVDR monitoring. The trade-off of the additional spotting and drying procedures is the relief of the stringent shipping and storage requirements for the fresh plasma specimens, which could be advantageous in certain circumstances.

#### DSS

DSS can be prepared by spotting and drying serum drops onto a filter paper card. While other dried spot analytes are often applied in HIVDRT, DSS usage is rarely reported, even though the suitability of DSS for HIVDRT has been confirmed [48,49,50,51]. HIV PR and RT gene amplification was achieved from >86% of DSS specimens with VL >10,000 copies/mL [49]. DSS showed a good consistency under conditions representative of field conditions [48]. These support DSS as an alternative specimen for scaled HIVDR surveillance or even centralized HIVDR monitoring tests in RLS. Similar to DPS, the lack of a more stable HIV proviral DNA component in DSS compared to DBS results in a lower success rate of HIVDR typing. However, the DSS-based HIVDR profiling is expected to be comparable with plasma or serum. Strategies that may improve the long-term HIV viral RNA stability in DPS and DSS specimens would significantly improve the suitability of such analytes for more broad HIV molecular assays, including HIVDRT. In addition, a modified experimental design with shorter amplicons also enhances the robustness of DSS or DPS-based HIVDRT [52].

#### 2.5.2. Dried Analytes Collected with the Newer Generation of Devices

Besides the filter paper card, a newer generation of dried specimen collection devices has been developed since the early 2010s. Two exemplary product series of this category, HemaSpot^TM^ and ViveST^TM^, are described below:

#### HemaSpot^TM^

Hemaspot^TM^ is a product series designed explicitly for dried blood specimen collection offered by Spot on Sciences (San Francisco, CA, USA), founded in 2010 (https://www.spotonsciences.com/, accessed on 20 March 2022). The HemaSpot HF device has been tested in HIVDR assays. The HemaSpot HF device is a moisture-tight cartridge containing a circular chamber. A spoked absorbent filter paper pad, a descant ring, and an application disk are layered from the bottom up. Once the fluid drops are loaded, the cartridge can be closed immediately. The built-in desiccant dries the sample in minutes, and the sample is then ready for shipment. Samples collected with HemaSpot devices closely mimic the DFPAs while easing the requirement for additional drying procedures and holding up to 200 μL of original liquid specimens. The moisture-tight design and the tamper-resistant latch on the cartridge also help minimize the ambience’s impact and retain the stability of the dried analyte it holds. Upon analysis, each spoke on the dried fluid pad can be plucked individually for multiple assays without punching, minimizing the risk of cross-contamination.

Dried blood analytes collected with HemaSpot have been applied as an alternative to DBS for serologic tests for several human viral pathogens [53,54,55,56]. While HemaSpot appears to be a promising technology, data on the usage of HemaSpot specimens for HIV molecular assays are scarce. Hirshfield et al. first confirmed the feasibility of using self-collected HemaSpot blood specimens for HIV VL monitoring [57]. Brooks et al. pioneered applying dried whole blood prepared with HemaSpot devices in HIVDRT in 2016 [58]. They evaluated the performance of HemaSpot specimens prepared from either fresh blood at various VL dilutions and a storage time up to 4 weeks at room temperature, or thawed frozen whole blood, both at 100 μL. For all specimens at VL >1000 copies /mL, HIV typing was successful in 79% and 58% of HemaSpot specimens prepared from fresh and frozen-thaw blood, respectively. The genotyping success rates improved significantly with a shorter storage period and higher VLs. The HIV PR and RT gene sequences derived from HemaSpot specimens show >96% identity compared to those from matching plasmas. In addition, the HIVDR profiling concordance between the paired HemaSpot specimens and plasma was determined as 86% [58]. Considering the intrinsic differences between plasma and DBS analytes described above, such discrepancies are expectable. Nonetheless, this study showed that the HemaSpot is a promising dried blood sample collection technology that may promote expanded HIVDRT in RLS.

With the scarcity of follow-up studies on this technology, the findings from this pilot study remain to be confirmed by more comprehensive evaluations regarding its sensitivity, accuracy, and consistency. These may include verifying the findings from Brooks’ study on dried whole blood and assessing the performance of dried plasma or serum specimens collected with the HemaSpot device in HIV molecular assays. Notably, the HemaSpot SE product is designed to separate and dry the cellular components and serum onto the same absorbent membrane, which may serve different analysis needs targeting serum/plasma or cellular components of blood with one analyte. Comprehensive studies on the suitability of specimens from HemaSpot SE device for HIV molecular assays remain to be conducted.

#### ViveST^TM^

ViveST^TM^, formerly called SampleTanker^TM^ (ST), is another dried sample storage and transportation device marketed by ViveBio Scientific (Alpharetta, GA, USA) since 2013 (https://vivebio.com/, accessed on 15 June 2022). The ST tubes have a proprietary, non-paper-based absorbent matrix attached to the tube cap and a descant block at the bottom. The biological substance, such as proteins and nucleic acids, in the original liquid specimens can be retained on the matrix while the water part is evaporated during the drying process. The matrix can be rehydrated for downstream lab assays using molecular-grade water, and the reconstituted sample can then be recovered. Compared to 50~75 μL per DFPA spot and ~200 μL for the HemaSpot device, up to 1.5 mL of biological fluid can be loaded and dried onto the ST matrix and then stored or transported at ambient temperature. It is a revolutionary solution for collecting, storing, and transporting liquid specimens from the field. It aims to expand the decentralized collection of any liquid biological samples, including blood, plasma, serum, and other body fluid analytes.

Dried plasma specimens collected with the ST device have been validated for VL assays for HIV, hepatitis B virus (HBV), and hepatitis C virus (HCV) [59,60,61,62], implying the application value of this new device in viral molecular assays. Lloyd et al. first reported ST application in HIVDRT during the XIII International HIV Drug Resistance Workshop in 2004 [60]. While lower VL readouts were obtained consistently from the ST plasma compared with frozen plasma, which was expected, the HIVDR mutations identified from the two compared analytes were concordant for all examined HIV-1 gene fragments. These observations were confirmed in an expanded study by this research group, which further demonstrated that the HIV viral RNA in ST plasma retained good integrity throughout the eight weeks of storage at 23 °C, 37 °C, and −80 °C [59].

In contrast, less optimistic findings were reported by Diallo et al., who assessed the application of the ST device for HIVDRT in RLS. They collected the whole blood or plasma specimens using the ST device and stored them at ambient temperature for 2 or 4 weeks. The performance of these two new analytes were compared against frozen plasma. Compared to 96% from frozen plasma specimens, both of the two new analytes performed poorly with significantly lower genotyping rates (48.98% and 42.85% for ST whole blood specimens stored for 2 and 4 weeks; 36% and 36% for ST plasma specimens stored for 2 and 4 weeks, respectively). Although the nucleotide sequence identities and the HIVDR profiles are highly concordant with the matching frozen plasmas for the successfully genotyped specimens, the low amplification rates from both ST specimens suggest that the ST device may not be ideal for HIVDRT sample collection in RLS [63]. Similar conclusions were also drawn from a study by Kantor et al. in which the performance of two ST processed specimen types (ST^TM^-plasma (STP) and ST^TM^-blood (STB)) in HIV genotyping were assessed [64]. The HIVDRT success rates using STP and STB in the Kantor study were 32% and 12%, compared to 82% from matching frozen plasmas [64]. While the specimens in these studies varied, the unfavorable outcomes from the two newer studies raise concerns over the preservation of HIV RNA/DNA integrity in the samples collected with ST tubes [63,64]. The further assessment of ViveST analytes for HIVDRT is warranted.

## 3. Application Considerations on HIVDRT Analytes

As described above, the quantity and quality of the HIV viral contents vary significantly among different analytes. The suitability of these analytes for the HIVDRT differ. The sensitivity, consistency, and accuracy vary considerably among the analytes due to the distinct nature of clinical analytes and the integrity of the HIV RNA or DNA templates they contain. Data collected from different analytes may hold differing values as applied to subsequent HIVDR interpretation. While plasma is the preferred analyte, it is not always available, especially in RLS, and alternative analytes are often required.

The suitability of an analyte for HIVDRT primarily depends on the resource availability for sample collection and transfer and downstream data application. One factor often neglected in HIVDRT analyte selection is the requirements from the downstream experimental procedures and data interpretation, which may differ significantly. Genotypic HIVDRT examines the presence of HIV DRMs either individually by allele-specific assays or collectively by sequencing using Sanger sequencing or NGS. Allele-specific assays target narrow viral genetic regions on which the impact from HIV RNA/DNA degradation is minimal. This is especially advantageous when a poorer HIV template quality is expected, such as the DFPAs and other dried analytes. In contrast, sequencing-based HIVDRT usually requires longer templates, making them more susceptible to HIV RNA/DNA degradation. One strategy to mitigate the limitation of degraded analytes, such as DFTAs, for HIVDRT is to implement modified protocols that generate shorter PCR amplicons or sequencing libraries. Compared to HIV RNA, a higher PCR amplification and sequencing success rate are expectable if DNA extracts from PBMC are used, especially when the VL is low. If targeting longer HIV genomic region(s) by long-range PCR or long-template NGS sequencing, fresh plasma/serum specimens with minimal HIV RNA/DNA degradation or HIV DNA extracts from PBMCs are always preferred.

## 4. Conclusions

In conclusion, although many analytes can be applied for HIVDR genotyping, their performance varies. Each analyte has its pros and cons from practicability and applicability perspectives. No single analyte could satisfy all requirements and applications. The suitability of an analyte for HIVDRT depends on: (1) the resource availability and accessibility; (2) the target viral population (circulating cell-free HIV viral particle vs. cell-associated HIV provirus); (3) the ease and convenience of the specimen acquisition, transportation, and storage; (4) the expected assay sensitivity, precision, and reproducibility; and (5) the downstream application (i.e., clinical monitoring vs surveillance) of the data obtained from the HIVDR assay. The optimal analyte selection always relies on the trade-off between test accountability and logistical capacity. A combined application of all such analytes is inevitable when varied application needs and requirements are present. Although their performance varies, all analytes contribute to the enhanced HIVDR management in unique ways, especially in RLS.

## Figures and Tables

**Table 1:** Varied analytes applied in HIV drug resistance test.

Specimens	Specimen Collection/Preparation	Applications	Pros	Cons
Plasma	The supernatant harvested after centrifugation of anti-coagulated whole blood.	Conventional analyte for HIVDRT. Suitable for HIVDRT serving all relevant clinical, surveillance, and research needs.	Representing actively circulating HIV population. Maximal recovery of cell-free viral RNA in blood. Low RNA degradation/high template integrity. Suitable for varied assays.	Needs for phlebotomy, centrifugation, and low-temperature transportation and storage. Poor HIV amplification when VL is low.
Serum	The fluid left after natural clotting of whole blood.	Suitable for HIVDR test serving all relevant clinical, surveillance and research needs. Remnant sera from diagnosis often used in HIVDR surveillance or research projects.	Closest substitute to plasma. Representing actively circulating HIV population. No need for centrifuging device. Suitable for varied assays.	Needs for phlebotomy, low-temperature transportation and storage. Lower template concentration than plasma. Poor HIV amplification when VL is low.
Whole blood	Anti-coagulated whole blood collected via venipuncture.	Depending on the nucleic acid extracts used, it supports HIVDR analysis of circulating HIV viruses (RNA), archival proviruses (DNA), or general (TNA).	Covering circulating and integrated viruses. No need for centrifugation device. Short-term storage at ambient is acceptable. Good HIV amplification even when VL is low.	Needs for phlebotomy. Discordant HIVDR profiling to plasma. Not ideal for clinical HIVDR monitoring. Poor reproducibility.
PBMCs	Mononuclear cells isolated from anti-coagulated whole blood by density gradient centrifugation.	DNA from PBMCs is occasionally used for HIVDRT, primarily in research projects. Substitute when RNA test is not feasible. Retrospective HIVDR analysis in which the order of DRM occurrence is less a concern.	Proviral DNA in PBMCs is stable. High HIV amplification rates from patients with even undetectable plasma VL.	Needs for phlebotomy. Discordant HIVDR readout to plasma and limited reflection on circulating HIV population, limiting its value for clinical monitoring.
**Dried filter paper analytes**				
DBS	Spotting and drying blood drops onto filter paper cards.	Applied primarily in HIVDR surveillance testing and research studies, rarely for clinical monitoring.	Easy to collect, transport, and store. Good HIV amplification if TNA is applied. No strict requirement for venipuncture. Suitable for pediatric patients.	Low sensitivity due to small volume and viral RNA degradation. Discordant HIVDR profiling to plasma, not suitable for clinical monitoring.
DPS	Spotting and drying plasma drops onto a filter paper card.	Applicable for clinical monitoring when the VL is high, i.e., prior to ART initiation. HIVDR surveillance testing or research use.	Closely mimics plasma in representing circulating virus. Concordant to plasma for HIVDR profiling, if successfully genotyped.	As compared to DBS: Lower viral RNA integrity, lower HIV amplification; Shorter storage, lower ambient temperature, and shorter transportation is required; Needs for phlebotomy.
DSS	Spotting and drying serum drops onto a filter paper card.	Occasionally applied in surveillance testing. Attempted for centralized HIVDRT in RLS.		
**Dried analytes collected with the newer generation of devices**				
HemaSpot	Loading and drying blood drops onto a HemaSpot device.	Research use only thus far. May facilitate HIVDRT in RLS.	Easy to collect, transport, and store. No strict requirement for venipuncture.	Low sensitivity likely due to suboptimal viral RNA integrity. Preservation of viral RNA/DNA remains to be better determined. More validation studies needed before broader adoption in HIVDRT.
ViveST^TM^	Loading and drying liquid specimen onto ViveST matrix.	Research use only thus far. May facilitate HIVDRT in RLS.	Easy to collect, transport, and store. Applicable for different liquid specimens. Holds larger volume of liquid.	
